# Prediction of Joint Angles Based on Human Lower Limb Surface Electromyography

**DOI:** 10.3390/s23125404

**Published:** 2023-06-07

**Authors:** Hongyu Zhao, Zhibo Qiu, Daoyong Peng, Fang Wang, Zhelong Wang, Sen Qiu, Xin Shi, Qinghao Chu

**Affiliations:** 1 Key Laboratory of Intelligent Control and Optimization for Industrial Equipment of Ministry of Education, Dalian University of Technology, Dalian 116024, Chinawangzl@dlut.edu.cn (Z.W.); qiu@dlut.edu.cn (S.Q.);; 2 School of Control Science and Engineering, Dalian University of Technology, Dalian 116024, China; 3 Neurology Department, Dalian Municipal Central Hospital, Dalian 116024, China; 13019422638wf@163.com

**Keywords:** electromyography, cuckoo search, machine learning, noise reduction, joint angle prediction

## Abstract

Wearable exoskeletons can help people with mobility impairments by improving their rehabilitation. As electromyography (EMG) signals occur before movement, they can be used as input signals for the exoskeletons to predict the body’s movement intention. In this paper, the OpenSim software is used to determine the muscle sites to be measured, i.e., rectus femoris, vastus lateralis, semitendinosus, biceps femoris, lateral gastrocnemius, and tibial anterior. The surface electromyography (sEMG) signals and inertial data are collected from the lower limbs while the human body is walking, going upstairs, and going uphill. The sEMG noise is reduced by a wavelet-threshold-based complete ensemble empirical mode decomposition with adaptive noise (CEEMDAN) reduction algorithm, and the time-domain features are extracted from the noise-reduced sEMG signals. Knee and hip angles during motion are calculated using quaternions through coordinate transformations. The random forest (RF) regression algorithm optimized by cuckoo search (CS), shortened as CS-RF, is used to establish the prediction model of lower limb joint angles by sEMG signals. Finally, root mean square error (RMSE), mean absolute error (MAE), and coefficient of determination (R2) are used as evaluation metrics to compare the prediction performance of the RF, support vector machine (SVM), back propagation (BP) neural network, and CS-RF. The evaluation results of CS-RF are superior to other algorithms under the three motion scenarios, with optimal metric values of 1.9167, 1.3893, and 0.9815, respectively.

## 1. Introduction

Recently, wearable exoskeleton technology has been continuously developed [1,2]. In real life, it is mainly used in medical rehabilitation, and has played an important role in assisting stroke and amputation patients to walk. Patients can complete a great deal of repetitive physiological gait training with mechanical lower limbs, so as to re-establish the correct movement pattern, improve the quality of life, and participate in daily activities like healthy people [3,4].

Human motion intention recognition technology is a method to predict human motion patterns through various sensing systems. This technology has attracted increasing attention from all over the world and has important applications in many fields [5]. Zhao et al. [6] collected human gait data through wearable inertial sensors, and hidden Markov models and neural networks were combined to identify gait phases. Zhao et al. [7] proposed a gait reconstruction method based on a gradient descent algorithm and a gait evaluation method based on weighted dynamic time warping, which can quantify the abnormalities of hemiplegic gait and monitor the rehabilitation process of patients’ walking ability. Qiu et al. [8] used a wearable gait analysis system to capture the limb motions and reconstructed a 3D human body model with high precision. Qiu et al. [9] also proposed an automatic phase segmentation method for kayak rowing. The results show that the combination of arm trunk joint angles and a support vector machine algorithm can better perform the task of kayak paddling phase segmentation with an accuracy of 98.1%. The above methods are mainly based on inertial data, which can only reflect the current state of the motion but cannot predict the motion in advance [10].

Surface electromyography (sEMG) signal is a superposition of motor unit action potentials in time and space. It is a physiological signal emitted by the activity of muscle neurons during active movement of the human body [11], which reflects neuromuscular activity to a certain extent [12]. sEMG signal has the advantages of easy extraction, easy identification, non-invasiveness, etc. [13,14]. More importantly, sEMG signals appear before visible motion, and contain rich information about human motion, and so have great advantages as a medium for perceiving human–computer interactions [15]. Therefore, it is possible to use sEMG signals to predict the joint angles of human lower extremities, and the joint angle prediction results can be used as input signals to control the exoskeleton, so as to determine the subject’s movement intention in advance [16,17].

Triwiyanto et al. [18] proposed a new EMG-based method for elbow joint angle prediction, which used time-domain features, zero-crossing, and a Kalman filter. The ranges of root mean square error (RMSE) and Pearson correlation coefficient (CC) were 6.9° to 17.5° and 0.93 to 0.99, respectively. Liang et al. [19] combined a nonlinear autoregressive (NARX) model to create a Gaussian process autoregressive model for the knee angle prediction of sEMG signals. The mean normalized root mean square errors (NRMSEs) of the model predictions for the left and right legs were 0.0063 ± 0.0081 and 0.0032 ± 0.0028, respectively. In addition, Chen et al. [20] used a deep belief network consisting of restricted Boltzmann machines to estimate the flexion/extension angles of hip, knee, and ankle joints. Raj et al. [21] constructed a NARX multilayer perceptron neural network model to predict elbow joint angles based on sEMG signals. Gautam et al. [22] used a long-term recursive convolutional network (LRCN) based on transfer learning for knee angle prediction, and the mean absolute error (MAE) of predicted joint angles was 8.1% and 9.2% for healthy subjects and subjects with knee lesions, respectively.

In this paper, the sEMG signal and inertial signal of human lower limbs are simultaneously collected by the sensors. The sensed data are used to build a mapping model of the sEMG signals and joint angles in three real scenarios: walking on flat ground, going upstairs, and walking uphill, so as to perform joint angle prediction. The main contributions of this paper are as follows:

(1) The human lower limb muscles are modeled using OpenSim, and the muscle sites for collecting sEMG signals are selected based on the correspondence between the tendon lengths of the muscles in each part of the limb and the knee joint angle.

(2) The sEMG noise is reduced by a noise reduction algorithm using a combination of wavelet thresholding and complete ensemble empirical mode decomposition with adaptive noise (CEEMDAN), and then the time-domain features are extracted.

(3) The Shimmer sensors are used to collect the inertial signals of human lower limbs while collecting the sEMG signals to obtain the quaternion, and then the angles of the knee and hip joints are calculated using the quaternion based on coordinate transformation. 

(4) The cuckoo search (CS) algorithm is used to optimize the random forest (RF), so as to construct a prediction model between the sEMG signal and the joint angle for joint angle prediction.

## 2. Data Acquisition and Processing

In this section, the inertial signal and sEMG signal of the lower limb were simultaneously collected by sensors. After filtering and denoising the collected raw data, the time-domain features of the sEMG were extracted, and the joint angles were calculated. The equipment and methods used are as follows.

### 2.1. Experimental Equipment

**Figure 1:**
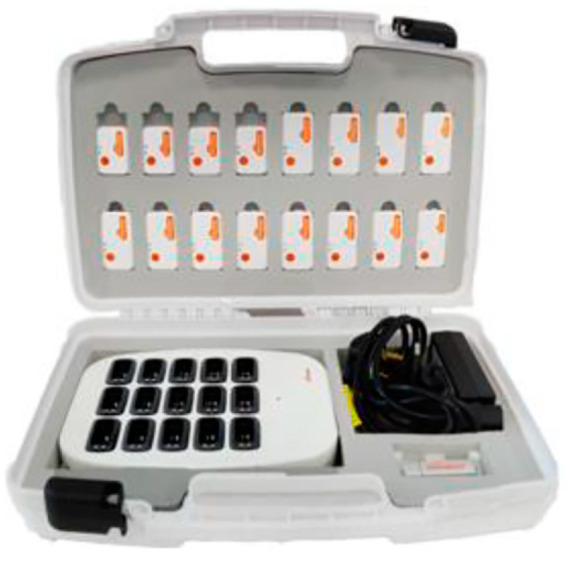
Consensys bundle development kit.

The Consensys bundle development kit is used in this paper for data correction, which is developed by the Shimmer technologies, Dublin, Ireland, and includes a complete set of Shimmer3 sensors, base, and software, as shown in Figure 1. Each Shimmer3 sensor can simultaneously measure the output of a triaxial accelerometer, a triaxial gyroscope, a triaxial magnetometer, and a two-channel sEMG, which has dimensions of 65 mm × 32 mm × 12 mm, and a weight of 31 g. Some specifications of the Shimmer3 sensors are shown in Table 1.

### 2.2. Muscle Selection

**Figure 2:**
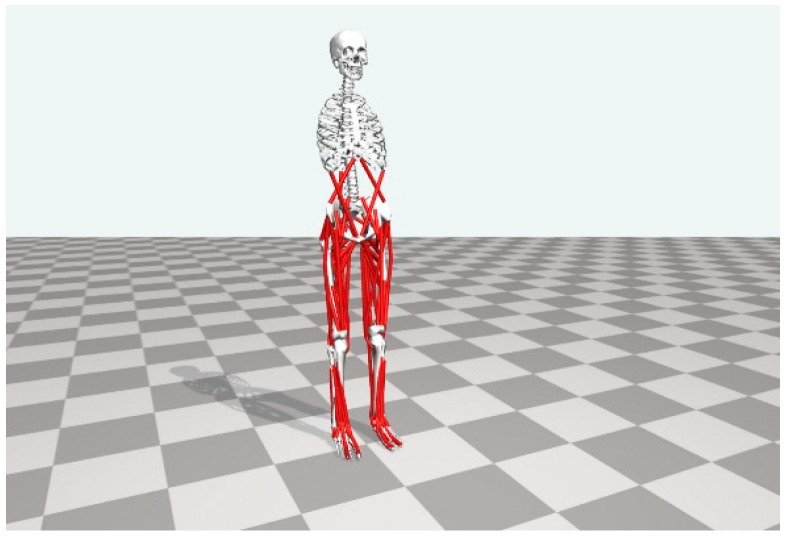
Model of human lower limb constructed by OpenSim software version 4.0.Beta.

OpenSim, a free software developed by the Stanford team, was used to address the problem of muscle modeling and simulation [23]. It has been applied to muscle modeling, motion simulation, and nervous system research. In this section, OpenSim is used to build a model of the human lower extremity, with 19 bones and 92 muscles, as shown in Figure 2, where the model has a height of 1.8 m and a weight of 75 kg. Note that the purpose of building the model is to observe the muscle parts of the human lower extremities, so it has little relationship with the model’s height and weight.

**Figure 3:**
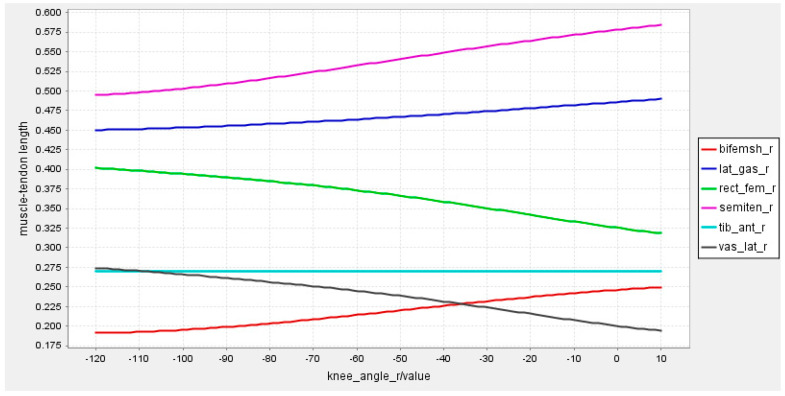
Relationship between tendon length and knee joint angle.

**Figure 4:**
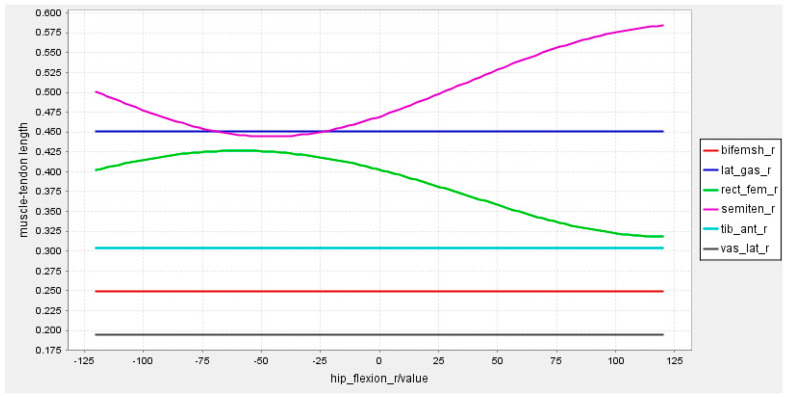
Relationship between tendon length and hip joint angle.

**Figure 5:**
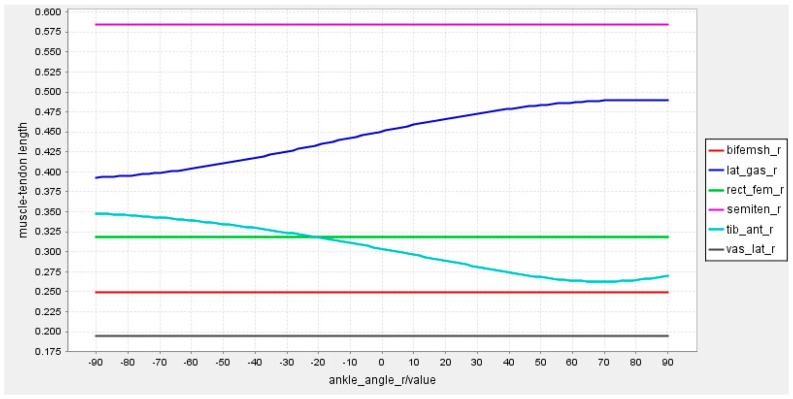
Relationship between tendon length and ankle joint angle.

As is known, too many measurement channels of sEMG can cause interference and reduce recognition performance [24]. Therefore, the selection of muscle sites for sEMG signals acquisition needs to consider the ease of acquisition as well as the active level of the muscles. According to the model constructed by the OpenSim software version 4.0.Beta, six muscles were selected for sEMG signal acquisition, i.e., rectus femoris, vastus lateralis, semitendinosus, biceps femoris, calf lateral gastrocnemius, and tibialis anterior. For each part of the lower limb, the relationship between the muscle−tendon length and the angles of the knee joint, hip joint, and ankle joint were established, respectively, as shown in Figure 3, Figure 4 and Figure 5.

From the joint angle and tendon length control charts, it can be seen that these six muscles work in concert during lower limb movements with different joint angle variations. These six muscles work in concert and the changes in tendon length can generate sEMG signals that reflect different movement states.

**Figure 6:**
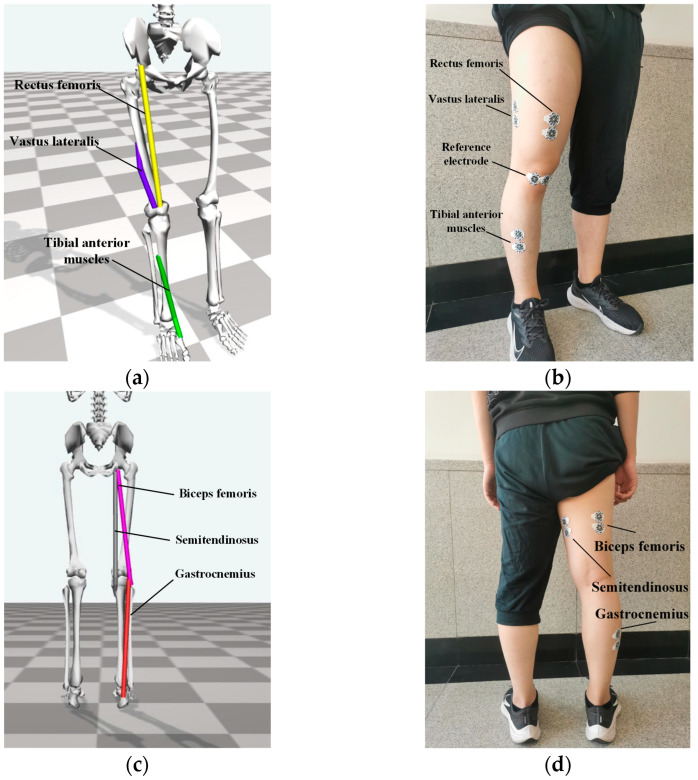
Comparison chart of selected muscle positions. (**a**) OpenSim frontal muscle sites; (**b**) human frontal muscle sites; (**c**) OpenSim back muscle sites; (**d**) human back muscle sites.

According to the muscle positions in the OpenSim software version 4.0.Beta, electrode patches were placed on the corresponding muscle parts of the subject’s lower limbs, as shown in Figure 6. The reference electrode for each sensor needs to be placed at the location where fewer lower limb muscles are present. In this experiment, the knee was chosen for the placement of the reference electrode.

### 2.3. sEMG Signal Filtering and Noise Reduction

The raw collected EMG signals often contain various noises, including power frequency noise, motion artifact noise, and electrocardiogram noise. As noise will mask the effective information in sEMG signals, it needs to be filtered out during data preprocessing [25]. Many noise reduction algorithms have been proposed for noise reduction in sEMG signals, such as wavelet thresholding, empirical modal decomposition (EMD), and ensemble empirical mode decomposition (EEMD). For noise reduction based on the wavelet thresholding method, the selection of the threshold value is very troublesome and affects the effect of noise reduction. For the noise reduction based on EMD decomposition, there exist endpoint effects and modal aliasing problems in the decomposition process. 

In this paper, considering the shortcomings of the above noise reduction methods, wavelet thresholding and CEEMDAN combination algorithm is used for noise reduction in the sEMG signals. Based on the CEEMDAN, the sEMG signal is decomposed to find the Intrinsic Mode Function (IMF) component. The correlation coefficient between the IMF and the sEMG signal is calculated to find out the high-frequency component that is dominated by noise, and the high-frequency component is processed by wavelet threshold noise reduction. The noise-reduced components are reconstructed with the unprocessed components to obtain the denoised signal.

Unlike the EMD and EEMD methods, the CEEMDAN method uses adaptive white noise in the decomposition to smooth out impulse interference and uses the property of averaged Gaussian white noise with a mean equal to zero to make the decomposition of the data more complete, thus effectively avoiding IMF aliasing within a certain range [26]. The specific algorithmic steps of CEEMDAN are as follows:

(1) A set of white Gaussian noise sequences are added to the original signal x(t) to form the new signal $x_{n}(t)$ as

(1) $$x_{n}(t)=x(t)+\varepsilon_{0}G_{i}$$

where t is the time index, i is the number of trials, $G_{i}$ is the Gaussian noise series with normal distribution, and $\varepsilon_{0}$ is the ratio of data versus noise to control the additional noise of the original data set.

(2) The first IMF component can be obtained by decomposing the sEMG signal using the conventional EMD method as

(2) $$IMF_{1}(t)=\frac{1}{k}\sum_{i=1}^{k}IMF_{1}^{i}(t)$$

where k is the number of times that Gaussian white noise with a mean of 0 is added to the original signal.

(3) The first residual $r_{1}(t)$ is calculated as

(3) $$r_{1}(t)=x(t)-IMF_{1}(t)$$

(4) To generate the *j*th pattern, the operator $E_{j}(\cdot )$ is defined first, then the new data sequence $r_{1}(t)+\varepsilon_{0}E_{1}(G_{i})$ is decomposed and the second IMF component is obtained using the EMD method as

(4) $$IMF_{2}(t)=\frac{1}{k}\sum_{i=1}^{k}E_{1}[r_{1}(t)+\varepsilon_{2}E_{1}(G_{i})]$$

(5) The *k*th remaining residue can be calculated for each of the remaining steps. The (*k* + 1)th IMF component is calculated by cycling through step 4, which is as follows:

(5) $$r_{j}(t)=r_{j-1}(t)-IMF_{j}(t)$$

(6) $$IMF_{j+1}(t)=\frac{1}{k}\sum_{i=1}^{k}E_{1}[r_{j}(t)+\varepsilon_{j}E_{j}(G_{i})]$$

(6) Step (5) is computed cyclically until the obtained residual is no longer able to be decomposed to obtain the final residual R(t) as

(7) $$R(t)=x(t)-\sum_{m=1}^{N}IMF_{m}(t)$$

where *N* is the number of decomposed IMFs.

(7) The CEEMDAN method is used to decompose the original signal *x*(*t*) to obtain *K* IMF components and a residual component as

(8) $$x(t)=\sum_{m=1}^{N}IMF_{m}(t)+R(t)$$

The steps of the wavelet thresholding and CEEMDAN sEMG signal denoising method are as follows: firstly, the IMF components from high frequency to low frequency are decomposed by CEEMDAN; secondly, the IMF components and the original sEMG signal are correlated, a suitable threshold is selected for the noisy components, and the sEMG signal is denoised by the wavelet thresholding algorithm; finally, the denoised components are reconstructed with the unprocessed components to obtain the denoised signal.

**Figure 7:**
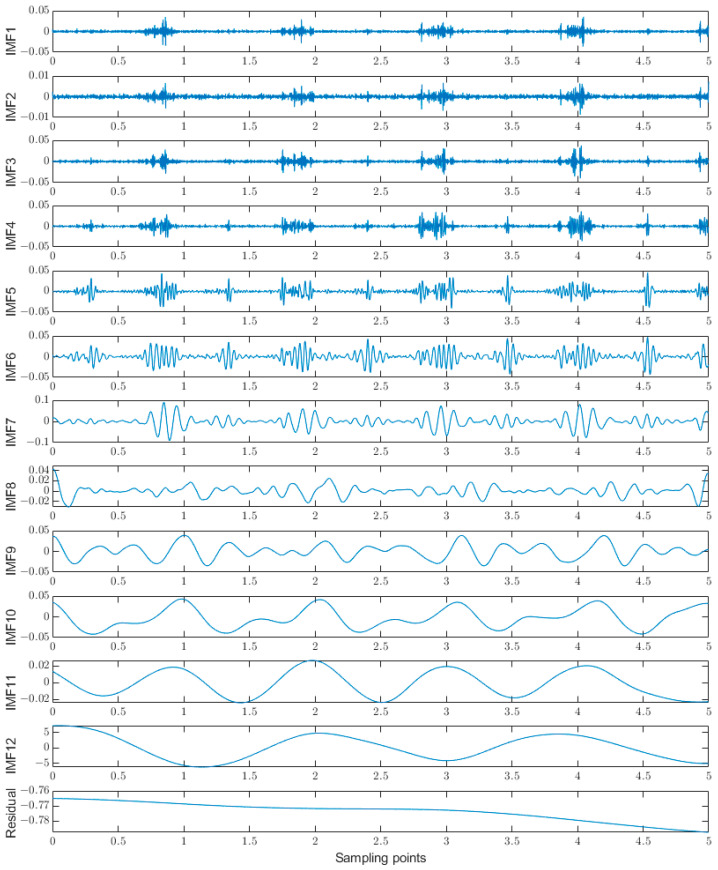
CEEMDAN decomposition.

In this section, the rectus femoris muscle during flat walking is taken as an example, and the raw sEMG signal is decomposed by CEEMDAN to obtain the IMF component and the residual component, as shown in Figure 7.

The resulting IMF quantities and the original signals are correlated to obtain the interrelationship numbers, as shown in Table 2.

**Figure 8:**
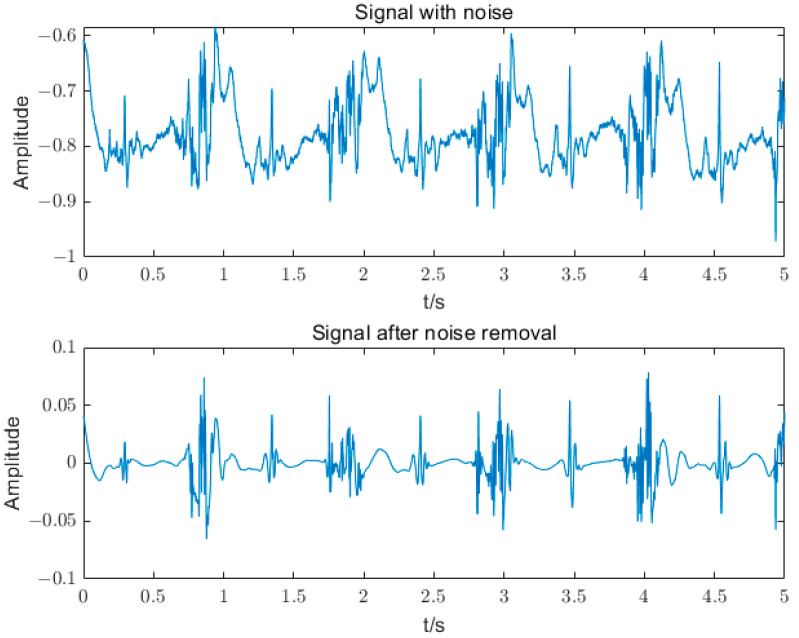
sEMG signals before and after noise reduction.

If the correlation coefficient of the high-frequency IMF component is less than 0.5, the IMF component contains noise. In this paper, the noisy IMF components are processed for noise reduction using heuristic thresholding combined with the db5 wavelet thresholding method. The baseline drift signal in the low-frequency IMF component is the noisy part of the sEMG signal. The IMF components with frequencies less than 5 Hz are filtered out by setting the threshold. Finally, the noise-reduced IMF component is reconstructed with the unprocessed component to obtain the denoised signal, and the results are shown in Figure 8.

The sEMG signals are generated based on the contraction of the muscle, so a valid sEMG signal should oscillate for y = 0 at equal amplitude. It can be seen that there is a significant baseline drift in the raw sEMG signals with many spikes, which are mixed with a large amount of noise. After noise reduction, most of the noise signals are eliminated, and the sEMG signals with more effective information are obtained.

In this paper, MSE is used as the evaluation index of the noise reduction performance [27], which can be calculated as

(9) $$MSE=\frac{\sum_{i=1}^{n}{\left(X_{e,i}-X_{r,i}\right)}^{2}}{n}$$

where *n* is the number of signals, $X_{e,i}$ is the original signal before noise reduction, and $X_{r,i}$ is the signal after noise reduction.

The wavelet thresholding method and CEEMDAN combined with the noise reduction method are compared with the Butterworth filtering method and wavelet thresholding noise reduction method. The comparative results for the rectus femoris muscle during flat walking are shown in Table 3, and the same for all muscle sites in different motion states.

From the results shown in Table 3, it can be seen that the wavelet thresholding method and CEEMDAN combined with noise reduction have a better noise reduction performance than other noise reduction methods.

### 2.4. sEMG Signal Feature Extraction

Feature extraction is an important process to extract the useful information hidden in EMG signals and remove the unwanted noise [28]. When preprocessing the sEMG signals, the sliding window method is used for signal segmentation. In this paper, the sliding window size is 10 ms.

The features extracted from sEMG signals are mainly time-domain features, frequency-domain features, and time–frequency-domain features. Among them, the most commonly used is the time-domain feature [29], which has the advantages of less complexity, low calculation cost, and good classification performance. In this study, four time-domain features were selected for each channel, including root mean square, variance, wavelength, and mean absolute value. The formulas for feature extraction are given below, where *N* is the size of the sliding window and *x* is the magnitude of the sEMG signal with a mean value of $\bar{x}$.

(1) Root Mean Square

(10) $$RMS=\sqrt{\frac{1}{N}\sum_{i=1}^{N}x_{i}^{2}}$$

(2) Variance

(11) $$VAR=\frac{1}{N-1}\sum_{i=1}^{N}{\left(x_{i}-\bar{x}\right)}^{2}$$

(3) Wavelength

(12) $$WL=\sum_{i=1}^{N-1}\left|x_{i+1}-x_{i}\right|$$

(4) Mean Absolute Value

(13) $$MAV=\frac{1}{N}\sum_{i=1}^{N}\left|x_{i}\right|$$

After obtaining the selected features, the data preprocessing of sEMG signals can be achieved.

### 2.5. Joint Angle Calculation

**Figure 9:**
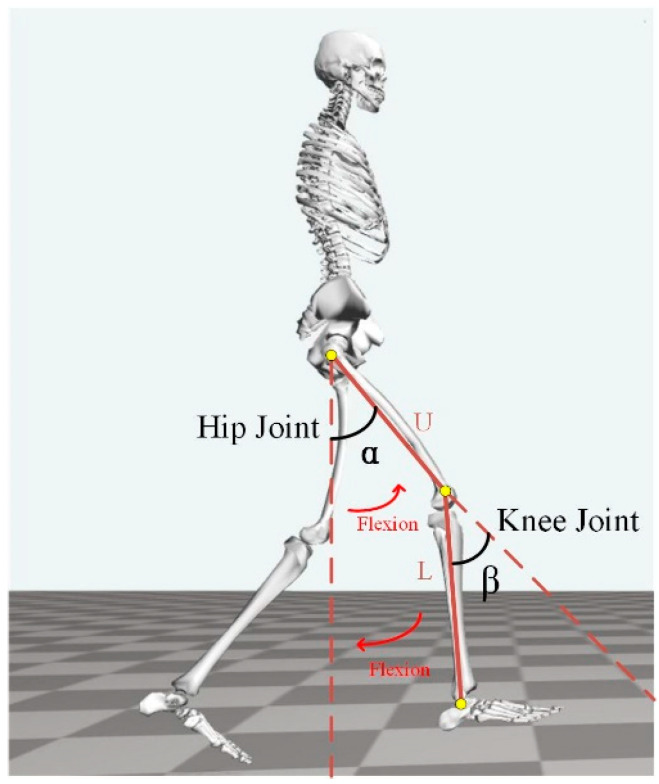
Definition of joint angles.

In this section, the knee and hip angles in the sagittal plane of the human body are estimated using the sEMG signals. The two joint angles are shown in Figure 9, which are zero when the body is in a neutral position. The detailed definition is as follows:

(1) The hip angle *α* is defined as the angle between the thigh U and its neutral position. The directions of hip flexion and extension are illustrated, respectively, in the figure, where flexion is positive and extension is negative.
(2) The knee joint angle *β* is defined as the supplementary angle of the angle between the thigh U and the calf L. Due to the constraints of the musculoskeletal structure of the human body, only the flexion angle of the knee joint can be measured.

**Figure 10:**
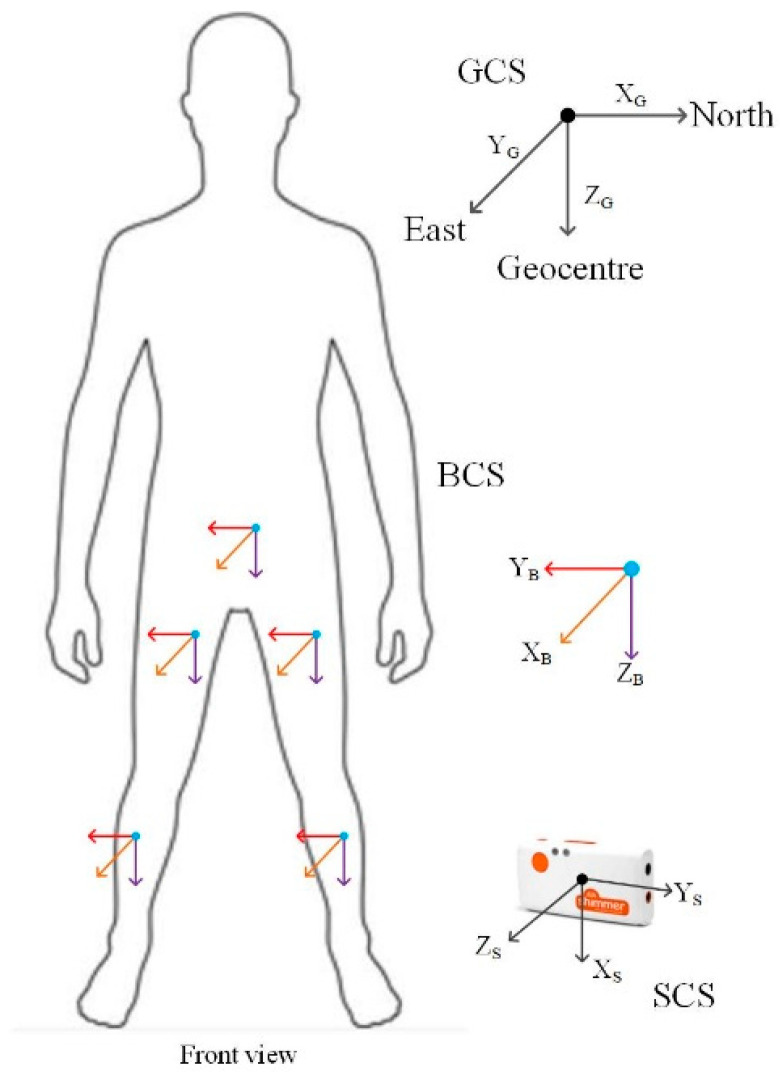
Definition of coordinate systems.

In order to calculate the joint angles, it is necessary to determine the coordinates of each limb segment in a unified coordinate system, as shown in Figure 10. Three right-handed coordinate systems are used in this paper, which are defined as follows:

(1) The global coordinate system (GCS or G for short) is defined as the local geographic coordinate system, whose X, Y, and Z axes point to the north, east, and center of the Earth, respectively.
(2) The body coordinate system (BCS or B for short) is fixed on the subject’s body and is defined at each joint to describe the relative position between the sensors and the limb.
(3) The coordinate system of the Shimmer3 sensor is defined as the sensor coordinate system (SCS or S for short).

In this paper, quaternions are used to describe the posture in different coordinate systems. The rotational relationship from BCS to SCS is assumed to be constant. At the beginning of the experiment, the subjects wore sensors on the lower extremity and stood still facing north for a few seconds so that the BCS and GCS coincided. The rotational relationship from BCS to SCS is obtained with the following equation, where * represents the conjugate quaternions.

(14) $$q_{B}^{S}=q_{B,init}^{S}=q_{G,init}^{S}={\left(q_{S,init}^{G}\right)}^{*}$$

The rotational relationship from BCS to GCS at moment *t* can be found from the obtained $q_{B}^{S}$, with the following equation, where ⊗ represents the product of the quaternions.

(15) $$q_{B,t}^{G}=q_{S,t}^{G}⊗q_{B}^{S}=q_{S,t}^{G}⊗(q_{S,init}^{G})$$

Suppose the vectors of the thigh, calf, and waist in BCS are *U_B_*, *L_B_*, and *W_B_*, respectively. Taking the thigh as an example, the rotation of the vectors from BCS to GCS can be obtained using $q_{B,t}^{G}$ through the following equation:

(16) $$U_{G,t}=q_{B,t}^{G}⊗U_{B}⊗{\left(q_{B,t}^{G}\right)}^{*}$$

The joint angles can be obtained by calculating the angles between the two vectors adjacent to them. The equations for the hip angle *α(t)* and knee angle *β(t)* at moment *t* are shown below.

(17) $$\alpha (t)=\arccos(\frac{U_{G,t}\cdot W_{G,t}}{\left\|U_{G,t}\right\|\times \left\|W_{G,t}\right\|})$$

(18) $$\beta (t)=\arccos(\frac{U_{G,t}\cdot L_{G,t}}{\left\|U_{G,t}\right\|\times \left\|L_{G,t}\right\|})$$

**Figure 11:**
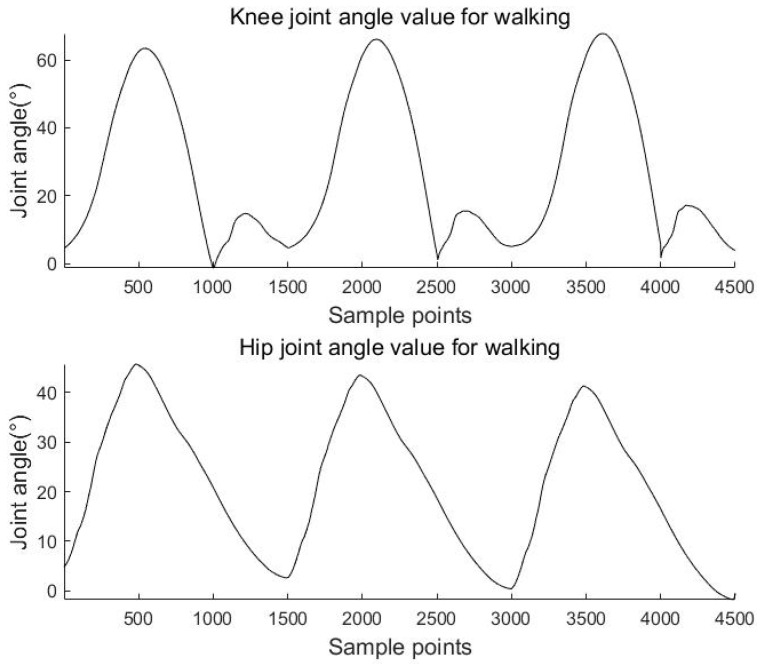
Knee and hip joint angles for walking.

**Figure 12:**
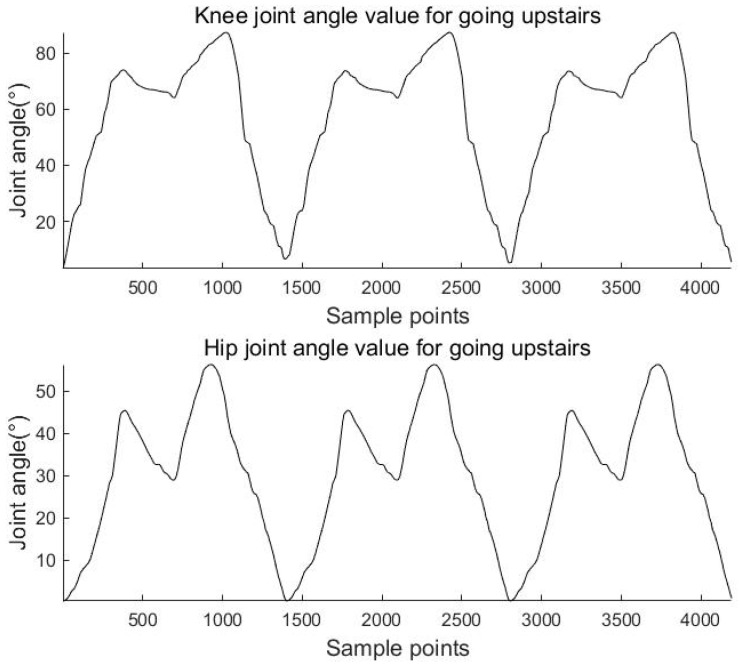
Knee and hip joint angles for going upstairs.

**Figure 13:**
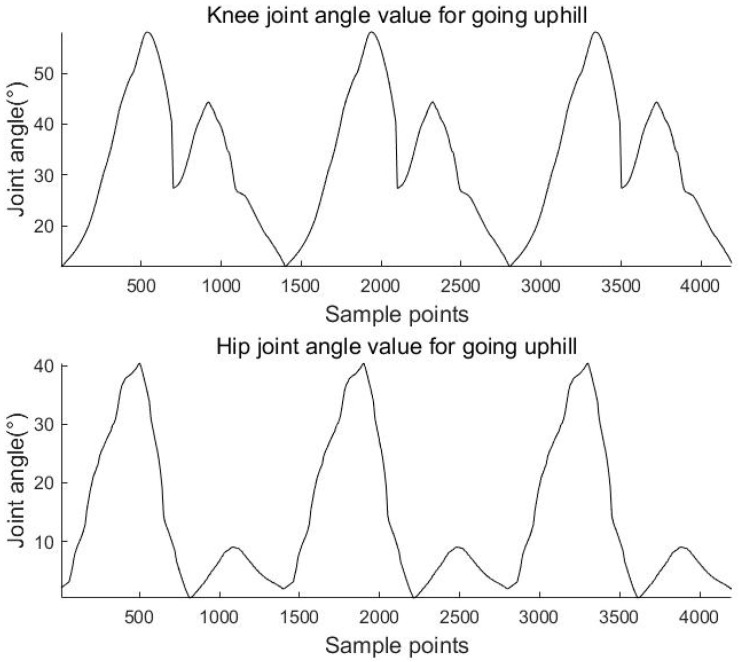
Knee and hip joint angles for going uphill.

The above equations are used to calculate the knee and hip angles for walking, going upstairs, and going uphill. The results are shown in Figure 11, Figure 12 and Figure 13.

## 3. Cuckoo-Search-Optimized Random Forest Regression

In recent years, the regression problem has usually been based on some nonlinear prediction algorithms, such as neural networks, support vector machines (SVM), and RF. RF is a supervised machine learning algorithm based on integration learning. Integrated learning is a type of learning where different types of algorithms or the same algorithm can be added multiple times to form a more powerful predictive model. RF combines multiple decision trees to produce prediction results and each decision tree is built with only a portion of samples and features, which not only makes it effective in preventing low accuracy in the presence of noise and outliers, but also does not drain the information from the sample data, ensuring the diversity of decision trees and greatly improving the generalization ability. The main parameters of the RF include the number of trees (n_estimators) and the maximum number of randomly selected features per node (max_features). In order to obtain a better RF model, the cuckoo search algorithm is used to optimize the RF in this paper.

**Figure 14:**
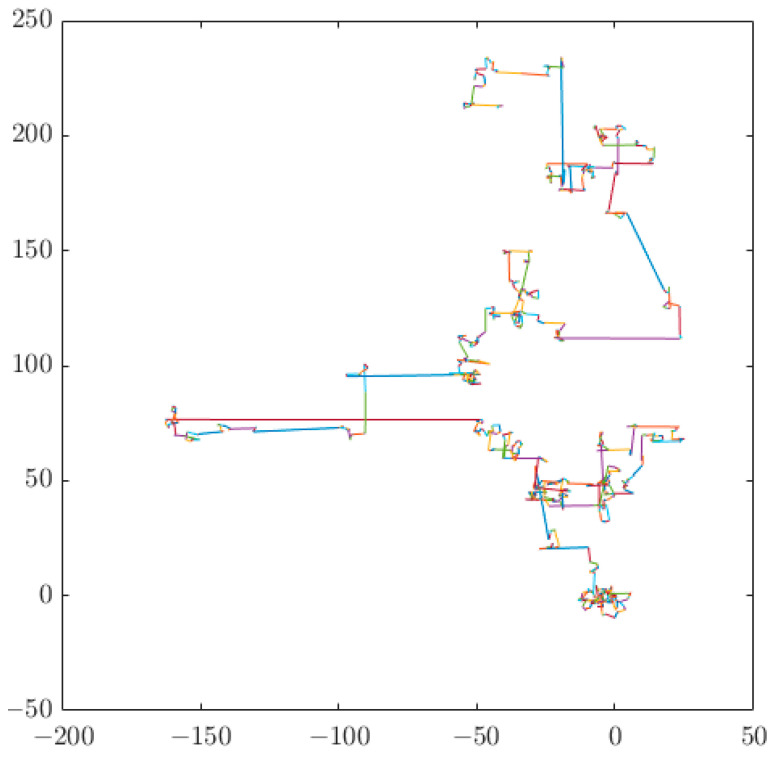
Lévy flight.

The CS algorithm, proposed by Yang et al. at the University of Cambridge in 2009, is a new intelligent optimization algorithm and a new meta-heuristic search algorithm, which has been used in many applications [30]. The idea of the CS algorithm is to solve the optimization problem based on the parasitic and juvenile characteristics of cuckoo birds and the overall optimization ability of Lévy flight. Parasitic brood rearing refers to the fact that cuckoos do not build nests during the breeding period, but rather place their eggs in the nests of similar birds and use these birds to reproduce and feed their offspring. Cuckoos seek out birds with similar incubation and brooding periods and quickly parasitize eggs while the bird is away, allowing the bird to raise their offspring instead [31]. Lévy flight is a random wanderer that balances local and global search by generating a certain number of long steps and a larger number of short steps, following a power-law step distribution with heavy tails. The method proposed by Mantegna in 1994 for solving random numbers with a positive-terrestrial distribution is mainly employed to generate random step lengths obeying a Lévy distribution, as shown in Equation (19) and Figure 14.

(19) $$s=\frac{u}{{\left|v\right|}^{\frac{1}{\beta }}}$$

where u and v are random numbers that conform to a normal distribution, i.e., $u∼N(0,\sigma^{2})$ and v∼N(0,1), and β takes a value in the range (0, 2) and generally 1.5.

(20) $$\sigma ={\left\{\frac{\Gamma (1+\beta )\sin(\frac{\pi \beta }{2})}{\beta \Gamma (\frac{1+\beta }{2})2^{\frac{\beta -1}{2}}}\right\}}^{\frac{1}{\beta }}$$

The specific steps of the CS algorithm to optimize RF are as follows:

(1) The number of nests and the number of iterations are set. One nest represents a set of parameters, which are n_estimators and max_features. Each set of parameters is randomly initialized before the start of the experiment.
(2) The criterion of comparison is chosen as fitness. In this paper, the RMSE is used as the value of fitness defined in Equation (21). The parameters in each nest are used to construct an RF algorithm separately to predict the lower limb joint angles. The predicted and true value adaptations of each algorithm are compared, and the nests corresponding to the parameters of the algorithm with the best adaptation are optimal.

(21) $$\mathrm{RMSE}=\sqrt{\frac{\sum_{i=1}^{n}{\left(X_{p,i}-X_{a,i}\right)}^{2}}{n}}$$

where *n* is the number of signals, $X_{p,i}$ is the observed value, and $X_{a,i}$ is the actual value.

(3) The optimal nest is retained and the other nests are updated by random wandering. The path of updating the bird nest location is as follows.

(22) $$x_{i}^{\left(t+1\right)}=x_{i}^{\left(t\right)}+\alpha ⊙Levy(\lambda );i=1,2,...,n$$

where $x_{i}^{\left(t\right)}$ is the value of the parameters of the *i*th nest in generation t, α is the step size factor whose value is usually 1, and Levy(λ) is the Lévy random search path, as shown in the following equation, where *s* denotes the size of the random step acquired by the Lévy flight.

(23) $$L(s,\lambda )∼s^{-\lambda },(1<\lambda \leq 3)$$

(4) The fitness value of the current nest is compared with the fitness value of the previous generation of nests. If the current nest is better, it replaces the previous generation of nests.
(5) A random number *R* between 0 and 1 is generated to be compared with the discovery probability *Pa*. If *R* > *Pa*, the nest is updated according to the set random wandering; if *R* < *Pa*, the nest is not updated.
(6) If the set number of iterations or the value of fitness is not reached, return to step (2).
(7) The final obtained global optimal solution is used as the parameter to construct the final RF algorithm in this paper.

## 4. Experiments and Results

In this study, the experiment was conducted outdoors. Subjects collected data using sEMG acquisition equipment and completed 3 movements in sequence, i.e., walking on flat ground, going upstairs, and going uphill. After data processing, the knee and hip angles during the 3 motion states could be estimated from the sEMG signals. The details are as follows.

### 4.1. Experimental Setup

**Figure 15:**
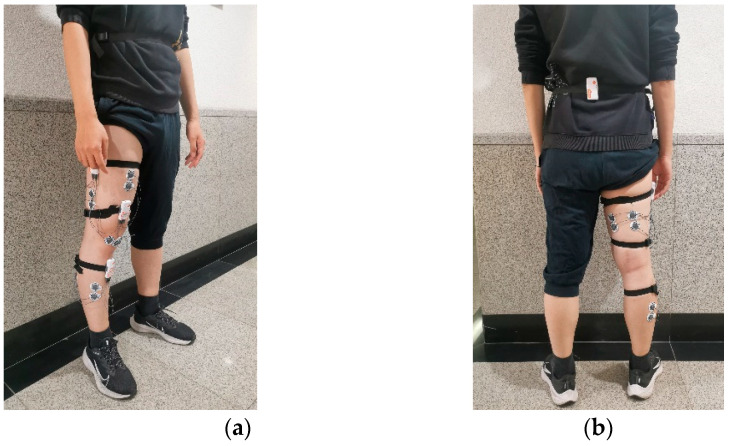
Diagram of sensor wearing: (**a**) front view of sensor; (**b**) back view of the sensor.

Data collection was performed using Shimmer3 sensors, each of which can collect nine-axis inertial data and 2 channels of sEMG signal. The sampling frequency was set to 1000 Hz, and 4 healthy subjects aged 22–35 years were recruited for data collection. According to Figure 6 and Figure 10, subjects wore one sensor on the waist for inertial data collection and 3 sensors on the right leg for sEMG data collection, as shown in Figure 15. The 2 sensors on the right leg were used to collect inertial data simultaneously, and in combination with the lumbar sensor to calculate knee and hip angles during motion.

**Figure 16:**
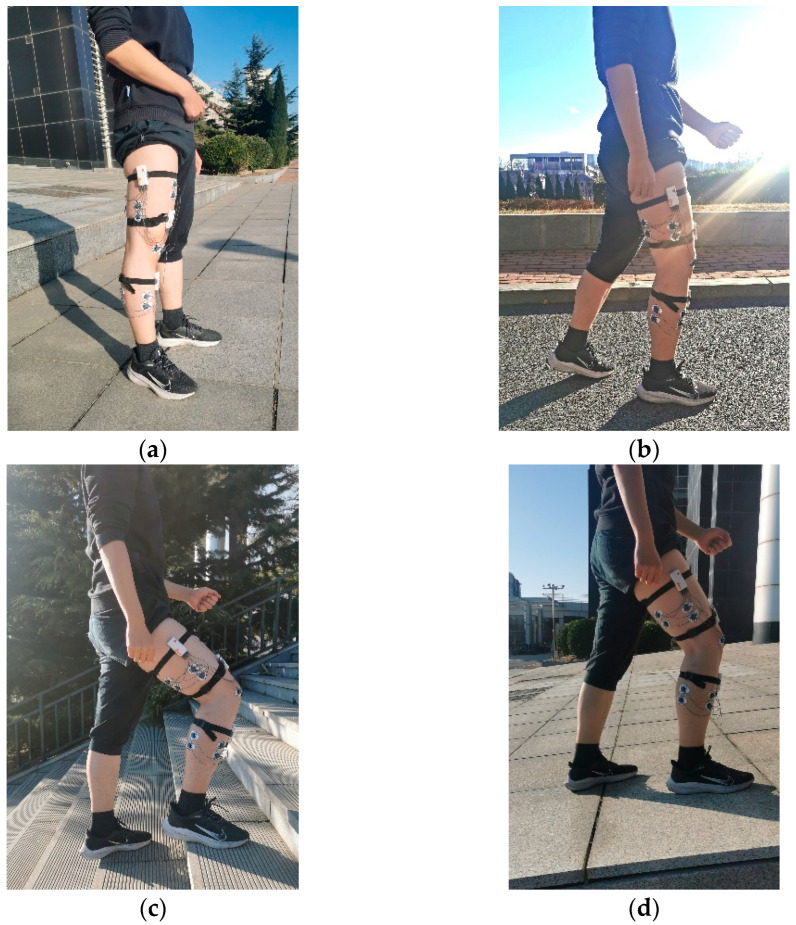
Comparison chart of selected muscle positions: (**a**) initial state; (**b**) walking; (**c**) going upstairs; (**d**) going uphill.

Each person collected data of 50 gait cycles per motion state, so there were 200 cycles of data per state. During the experiment, the subjects took a break every fifteen minutes to avoid muscle fatigue. The initial state and 3 motion patterns are shown in Figure 16.

### 4.2. Experimental Result

For the classification problem of sEMG signals, the sliding window size is permanently 50 ms–200 ms [32]. However, in this paper, the prediction of successive changes in joint angles was a regression problem. Thus, in order to obtain as many joint angles as possible in one exercise cycle, the sliding window size was set to be 10 ms for each cycle.

For the sEMG signals, the RMS, VAR, WL, and MAV features of each muscle were extracted using the sliding window, i.e., 6 muscles and 4 features in total, so each window constituted a 24-dimensional feature as the input signal of the algorithm. In addition, the average value of the joint angle of the lower limb at the same moment was calculated using the sliding window as the output of the algorithm. With a ratio of 4:1, 160 cycles were taken as the training set and 40 cycles as the test set. 

**Figure 17:**
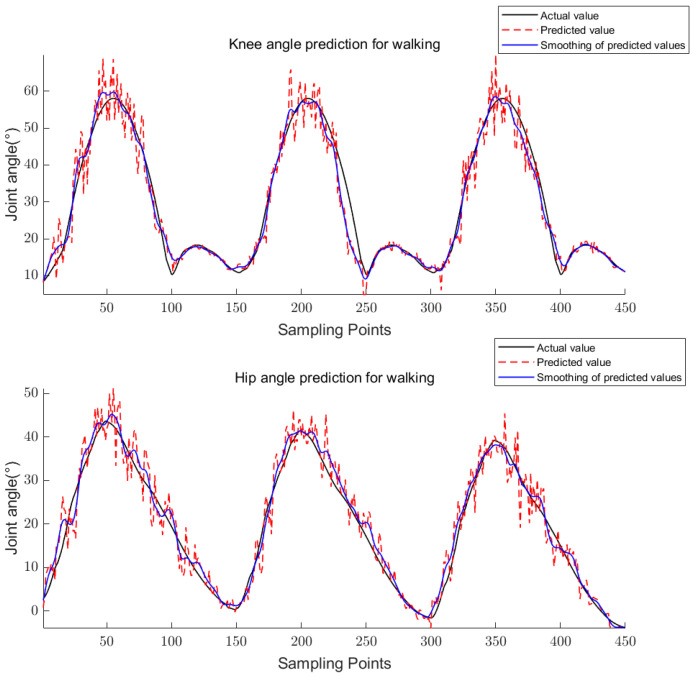
Prediction results of knee and hip joint angle for walking.

**Figure 18:**
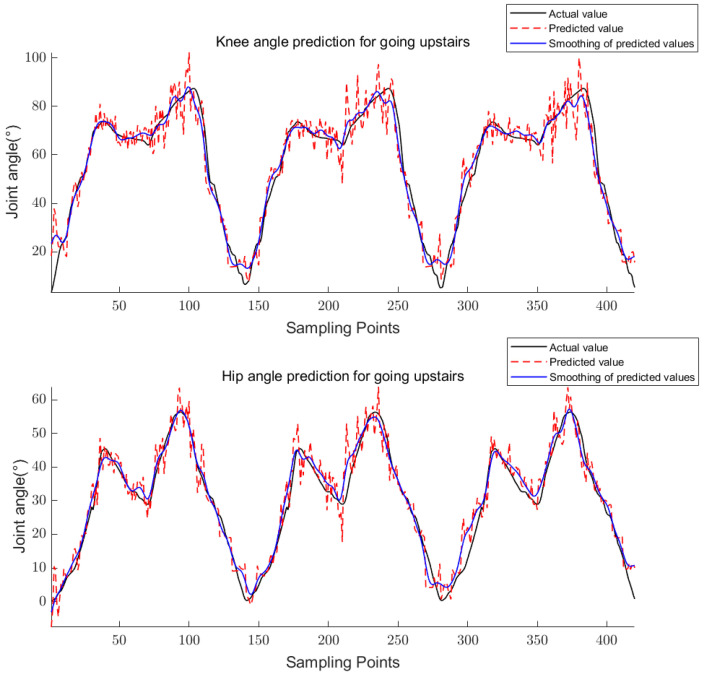
Prediction results of knee and hip joint angle for going upstairs.

**Figure 19:**
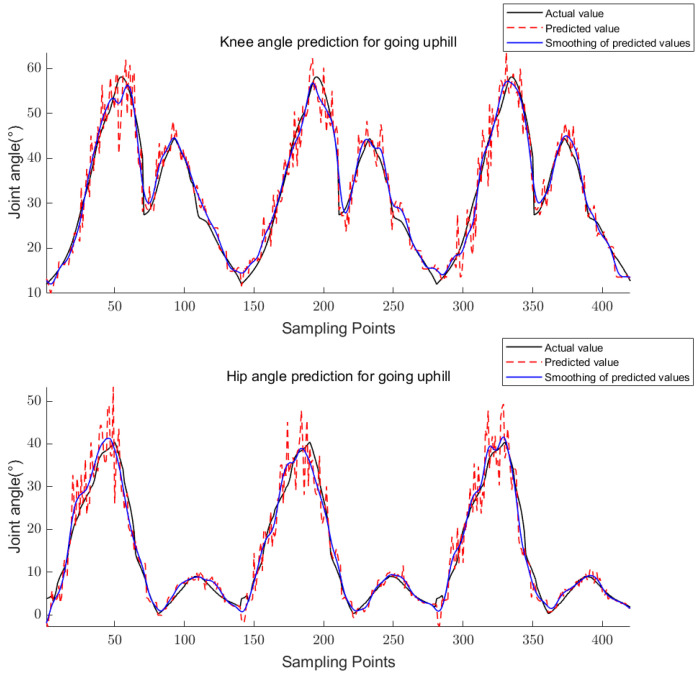
Prediction results of knee and hip joint angles for going uphill.

For training, the 24-dimensional features in the training set were taken as the input and the corresponding joint angle averages were taken as the output to obtain the trained CS-RF model; for prediction, the 24-dimensional features in the prediction set were taken as the input to obtain the predicted joint angles. The five-point triple smoothing method was used to process the joint angle prediction results and compare them with the actual joint angles. The final results are shown in Figure 17, Figure 18 and Figure 19. 

The CS algorithm parameters are mainly the number of nests and the number of iterations. The higher their values, the wider the search space as well as the higher the chance of search, but they also increase the computational overhead and computational time. In this paper, the number of nests of CS algorithm was set to 20, the number of iterations was set to 25, and the discovery probability was set to 0.25 through experiments.

In this section, three evaluation metrics are used to evaluate the results, i.e., RMSE, MAE, and coefficient of determination (R^2^) [33]. The formulas are as follows:

(1) RMSE

(24) $$\mathrm{RMSE}=\sqrt{\frac{\sum_{i=1}^{n}{\left(X_{p,i}-X_{a,i}\right)}^{2}}{n}}$$

(2) MAE

(25) $$\mathrm{MAE}=\frac{1}{n}\sum_{i=1}^{n}\left|X_{p,i}-X_{a,i}\right|$$

(3) R^2^

(26) $$R^{2}=1-\frac{{\sum_{i=1}^{n}\left(X_{p,i}-{\bar{X}}_{p}\right)}^{2}}{\sum_{i=1}^{n}{\left(X_{a,i}-{\bar{X}}_{a}\right)}^{2}}$$

where *n* is the number of signals, $X_{p,i}$ is the estimated value, ${\bar{X}}_{p}$ is the average of the estimated values, $X_{a,i}$ is the actual value, and ${\bar{X}}_{a}$ is the average of the actual values.

The results of the CS-RF algorithm are compared with those of the RF, SVM, and BP neural networks, as shown in Table 4, Table 5, Table 6, Table 7, Table 8 and Table 9.

From the above results, it can be seen that the evaluation index of CS-RF is significantly better than the remaining 3 algorithms under the three motion scenarios. The experimental results show that the prediction results of the CS-RF algorithm are superior compared to other algorithms. 

## 5. Conclusions and Future Work

sEMG signals are physiological signals that are closely related to human movement and play an important role in human–computer interaction. In this paper, a model for predicting the knee and hip angles of the lower limbs based on sEMG signals was developed. sEMG signals and inertial data of lower limbs were collected during walking, going upstairs, and going uphill. The algorithm combining wavelet thresholding and CEEMDAN was used for noise reduction first, then the time-domain features were extracted from the noise-reduced sEMG signals, and finally the CS-RF regression algorithm was used to predict the lower limb joint angles based on the pre-processed sEMG signals. As the RF algorithm is an integrated learning algorithm with a model constructed based on multiple decision trees, the prediction results were averaged by summing the predictions of all decision trees. Compared with individual decision trees, the RF algorithm can avoid the problems of overfitting and high model variance, and has better generalization ability. Compared with the neural networks commonly used in the current literature, CS-RF has good interpretability, which can be computed in parallel with high computational efficiency. Moreover, the optimized RF of CS has better generalization ability, which can better handle the input data and improve the prediction accuracy. RMSE, MAE, and R^2^ were used as evaluation metrics for algorithm comparison. The results show that the method proposed in this paper significantly outperforms other algorithms and provides help for the future control design of exoskeletons. The research will contribute to exoskeleton control design and may eventually improve user–machine interactions.

However, the study in this paper has some limitations. The predictions were offline and further studies are needed to predict joint angles in real time. The recruited subjects were healthy and there were no subjects with walking difficulties. In future work, we will investigate the real-time prediction of joint angles. In addition, patients with walking difficulties, e.g., hemiplegia, will be included in the experiments to enrich the data.

## Figures and Tables

**Table 1:** Specifications of Shimmer3 sensor.

	Accelerometer	Gyroscope	Magnetometer	EMG
Range	±16 g	±2000 dps	±49.152 gauss	±8000 mv
Sensitivity	1000 LSB/g	131 LSB/dps	667 LSB/gauss	-
Frequency	400 Hz	400 Hz	400 Hz	1000 Hz

**Table 2:** Correlation coefficient after CEEMDANN decomposition.

CEEMDAN Component	Correlation Coefficient
IMF1	0.1025
IMF2	0.1269
IMF3	0.1537
IMF4	0.1989
IMF5	0.2811
IMF6	0.3088
IMF7	0.4649
IMF8	0.3952
IMF9	0.4861
IMF10	0.6843
IMF11	0.5606
IMF12	0.0685

**Table 3:** MSE with different noise reduction methods.

Noise Reduction Method	MSE
Wavelet thresholding—CEEMDAN	0.6075
Wavelet thresholding	0.6092
Butterworth filtering	0.6093

**Table 4:** Comparative results of knee joint angle indexes during walking.

Algorithm	RMSE	MAE	R^2^
CS-RF	2.5855	1.6442	0.9771
RF	4.4622	3.7734	0.9297
SVR	5.5328	4.4292	0.8958
BP	8.3652	7.1166	0.7553

**Table 5:** Comparative results of hip joint angle indexes during walking.

Algorithm	RMSE	MAE	R^2^
CS-RF	1.9167	1.5363	0.9815
RF	2.8491	2.3740	0.9592
SVR	6.4977	5.4705	0.8685
BP	4.9283	4.1402	0.8762

**Table 6:** Comparative results of knee joint angle indexes during going upstairs.

Algorithm	RMSE	MAE	R^2^
CS-RF	4.0860	2.8571	0.9700
RF	6.3228	3.9982	0.9030
SVR	11.4990	8.2134	0.8824
BP	10.0615	6.9319	0.7544

**Table 7:** Comparative results of hip joint angle indexes during going upstairs.

Algorithm	RMSE	MAE	R^2^
CS-RF	2.7290	2.0576	0.9708
RF	3.7733	3.1052	0.9327
SVR	5.0233	3.8908	0.9179
BP	5.8566	4.9107	0.8287

**Table 8:** Comparative results of knee joint angle indexes during going uphill.

Algorithm	RMSE	MAE	R^2^
CS-RF	1.9336	1.5102	0.9794
RF	5.4610	4.5868	0.9511
SVR	9.5408	8.4636	0.8883
BP	8.3502	7.2950	0.8203

**Table 9:** Comparative results of hip joint angle indexes during going uphill.

Algorithm	RMSE	MAE	R^2^
CS-RF	1.9276	1.3893	0.9769
RF	3.2941	2.6668	0.9435
SVR	6.4199	5.4012	0.9067
BP	6.0689	5.1178	0.8249

## Data Availability

The data presented in this study are available on request from the corresponding author.

## References

[1] Liu D.X., Wu X., Du W., Wang C., Xu T. (2016). Gait Phase Recognition for Lower-Limb Exoskeleton with Only Joint Angular Sensors. Sensors.

[2] De Looze M.P., Bosch T., Krause F., Stadler K.S., O’sullivan L.W. (2016). Exoskeletons for industrial application and their potential effects on physical work load. Ergonomics.

[3] Dollar A.M., Herr H. (2008). Lower Extremity Exoskeletons and Active Orthoses: Challenges and State-of-the-Art. IEEE Trans. Robot..

[4] Fleming A., Stafford N., Huang S., Hu X., Ferris D.P., Huang H.H. (2021). Myoelectric control of robotic lower limb prostheses: A review of electromyography interfaces, control paradigms, challenges and future directions. J. Neural Eng..

[5] Qiu S., Zhao H., Jiang N., Wang Z., Liu L., An Y., Zhao H., Miao X., Liu R., Fortino G. (2022). Multi-sensor information fusion based on machine learning for real applications in human activity recognition: State-of-the-art and research challenges. Inf. Fusion.

[6] Zhao H., Wang Z., Qiu S., Wang J., Xu F., Wang Z., Shen Y. (2019). Adaptive gait detection based on foot-mounted inertial sensors and multi-sensor fusion. Inf. Fusion.

[7] Zhao H., Xu H., Wang Z., Wang L., Qiu S., Peng D., Li J., Jiang J. (2023). Analysis and Evaluation of Hemiplegic Gait Based on Wearable Sensor Network. Inf. Fusion.

[8] Qiu S., Wang H., Li J., Zhao H., Wang Z., Wang J., Wang Q., Plettemeier D., Bärhold M., Bauer T. (2020). Towards Wearable-Inertial-Sensor-Based Gait Posture Evaluation for Subjects with Unbalanced Gaits. Sensors.

[9] Qiu S., Hao Z., Wang Z., Liu L., Liu J., Zhao H., Fortino G. (2022). Sensor Combination Selection Strategy for Kayak Cycle Phase Segmentation Based on Body Sensor Networks. IEEE Internet Things J..

[10] Li K., Zhang J., Wang L., Zhang M., Li J., Bao S. (2020). A review of the key technologies for sEMG-based human-robot interaction systems. Biomed. Signal Process. Control.

[11] Reaz M.B.I., Hussain M.S., Mohd-Yasin F. (2006). Techniques of EMG signal analysis: Detection, processing, classification and applications. Biol. Proced. Online.

[12] Zhong B., Da Silva R.L., Li M., Huang H., Lobaton E. (2021). Environmental Context Prediction for Lower Limb Prostheses with Uncertainty Quantification. IEEE Trans. Autom. Sci. Eng..

[13] Wan D., Zhang L., Bai Y., Xie Y. Research on Identification Algorithm Based on ECG Signal and Improved Convolutional Neural Network. Proceedings of the International Conference on Computer Big Data and Artificial Intelligence.

[14] Laport F., Iglesia D., Dapena A., Castro P.M., Vazquez-Araujo F.J. (2021). Proposals and Comparisons from One-Sensor EEG and EOG Human-Machine Interfaces. Sensors.

[15] Artemiadis P. (2012). EMG-based Robot Control Interfaces: Past, Present and Future. Adv. Robot. Autom..

[16] Coker J., Chen H., Schall Jr M.C., Gallagher S., Zabala M. (2021). EMG and Joint Angle-Based Machine Learning to Predict Future Joint Angles at the Knee. Sensors.

[17] Cimolato A., Driessen J.J., Mattos L.S., De Momi E., Laffranchi M., De Michieli L. (2022). EMG-Driven Control in Lower LimbProstheses: A Topic-Based Systematic Review. J. Neuroeng. Rehabil..

[18] Triwiyanto T., Wahyunggoro O., Nugroho H.A., Herianto H. (2017). Evaluating the performance of Kalman filter on elbow joint angle prediction based on electromyography. Int. J. Precis. Eng. Manuf..

[19] Liang J., Shi Z., Zhu F., Chen W., Chen X., Li Y. (2021). Gaussian Process Autoregression for Joint Angle Prediction Based on sEMG Signals. Front. Public Health.

[20] Chen J., Zhang X., Cheng Y., Xi N. (2018). Surface EMG based continuous estimation of human lower limb joint angles by using deep belief networks. Biomed. Signal Process. Control.

[21] Raj R., Sivanandan K.S. (2017). Elbow joint angle and elbow movement velocity estimation using NARX-multiple layer perceptron neural network model with surface EMG time domain parameters. J. Back Musculoskelet. Rehabil..

[22] Gautam A., Panwar M., Biswas D., Acharyya A. (2020). MyoNet: A Transfer-Learning-Based LRCN for Lower Limb Movement Recognition and Knee Joint Angle Prediction for Remote Monitoring of Rehabilitation Progress From sEMG. IEEE J. Transl. Eng. Health Med..

[23] Seth A., Hicks J.L., Uchida T.K., Habib A., Dembia C.L., Dunne J.J., Ong C.F., Demers M.S., Rajagopal A., Millard M. (2018). OpenSim: Simulating musculoskeletal dynamics and neuromuscular control to study human and animal movement. PLoS Comput. Biol..

[24] Ai Q., Zhang Y., Qi W., Liu Q., Chen K. (2017). Research on Lower Limb Motion Recognition Based on Fusion of sEMG and Accelerometer Signals. Symmetry.

[25] Bisi S., De Luca L., Shrestha B., Yang Z., Gandhi V. (2018). Development of an EMG-Controlled Mobile Robot. Robotics.

[26] Colominas M.A., Schlotthauer G., Torres M.E. (2014). Improved complete ensemble EMD: A suitable tool for biomedical signal processing. Biomed. Signal Process. Control.

[27] Nagasirisha B., Prasad V.V.K.D.V. (2020). Noise Removal from EMG Signal Using Adaptive Enhanced Squirrel Search Algorithm. Fluct. Noise Lett..

[28] Phinyomark A., Phukpattaranont P., Limsakul C. (2012). Feature reduction and selection for EMG signal classification. Expert Syst. Appl..

[29] Jang G., Kim J., Choi Y., Yim J. (2014). Human shoulder motion extraction using EMG signals. Int. J. Precis. Eng. Manuf..

[30] Yang X.S., Deb S. (2013). Cuckoo search: Recent advances and applications. Neural Comput. Appl..

[31] Pandey A.C., Rajpoot D.S., Saraswat M. (2017). Twitter sentiment analysis using hybrid cuckoo search method. Inf. Process. Manag..

[32] Tigrini A., Al-Timemy A.H., Verdini F., Fioretti S., Morettini M., Burattini L., Mengarelli A. (2023). Decoding transient sEMG data for intent motion recognition in transhumeral amputees. Biomed. Signal Process. Control.

[33] Chicco D., Warrens M.J., Jurman G. (2021). The coefficient of determination R-squared is more informative than SMAPE, MAE, MAPE, MSE and RMSE in regression analysis evaluation. PeerJ Comput. Sci..

